# Web-Based Virtual Microscopy for Parasitology: A Novel Tool for Education and Quality Assurance

**DOI:** 10.1371/journal.pntd.0000315

**Published:** 2008-10-22

**Authors:** Ewert Linder, Mikael Lundin, Cecilia Thors, Marianne Lebbad, Jadwiga Winiecka-Krusnell, Heikki Helin, Byron Leiva, Jorma Isola, Johan Lundin

**Affiliations:** 1 Swedish Institute for Infectious Disease Control (SMI), Solna, Sweden; 2 Department of Microbiology, Tumor and Cellbiology (MTC), Stockholm, Sweden; 3 Biomedical Informatics Research Group, Folkhälsan Research Center and Institute of Clinical Medicine, University of Helsinki, HUCH Clinical Research Institute, Helsinki, Finland; 4 Division of Pathology, HUSLAB, Helsinki University Central Hospital, Helsinki, Finland; 5 Department of Microbiology, National University of Léon (UNAN), Léon, Nicaragua; 6 Institute of Medical Technology, University of Tampere, Tampere, Finland; Swiss Tropical Institute, Switzerland

## Abstract

**Background:**

The basis for correctly assessing the burden of parasitic infections and the effects of interventions relies on a somewhat shaky foundation as long as we do not know how reliable the reported laboratory findings are. Thus virtual microscopy, successfully introduced as a histopathology tool, has been adapted for medical parasitology.

**Methodology/Principal Findings:**

Specimens containing parasites in tissues, stools, and blood have been digitized and made accessible as a “webmicroscope for parasitology” (WMP) on the Internet (http://www.webmicroscope.net/parasitology).These digitized specimens can be viewed (“navigated” both in the x-axis and the y-axis) at the desired magnification by an unrestricted number of individuals simultaneously. For virtual microscopy of specimens containing stool parasites, it was necessary to develop the technique further in order to enable navigation in the *z* plane (i.e., “focusing”). Specimens were therefore scanned and photographed in two or more focal planes. The resulting digitized specimens consist of stacks of laterally “stiched” individual images covering the entire area of the sample photographed at high magnification. The digitized image information (∼10 GB uncompressed data per specimen) is accessible at data transfer speeds from 2 to 10 Mb/s via a network of five image servers located in different parts of Europe. Image streaming and rapid data transfer to an ordinary personal computer makes web-based virtual microscopy similar to conventional microscopy.

**Conclusion/Significance:**

The potential of this novel technique in the field of medical parasitology to share identical parasitological specimens means that we can provide a “gold standard”, which can overcome several problems encountered in quality control of diagnostic parasitology. Thus, the WMP may have an impact on the reliability of data, which constitute the basis for our understanding of the vast problem of neglected tropical diseases. The WMP can be used also in the absence of a fast Internet communication. An ordinary PC, or even a laptop, may function as a local image server, e.g., in health centers in tropical endemic areas.

## Author Summary

Here, we describe a novel tool to observe parasites by virtual microscopy on the Internet. Microscopy-based identification of parasites is the basis for both diagnostics and epidemiological assessment of parasite burden globally. Yet, quality assessment of diagnostic parasitology laboratories is difficult, as delivering identical educational specimens has been impossible. In this study, a series of parasite specimens on ordinary glass slides were digitized using a recently developed microscope scanner technique. Up to 50,000 images captured at high magnification are digitally stitched together to form a representation of the entire glass slide. These “virtual slides” digitized at a thousand-fold magnification can hold more than 60 gigabytes of data. Handling such large amounts of data was made possible because of efficient compression techniques and a viewing system adopted from the geospatial imaging industry. Viewing the samples on the Internet very much resembles, for example, the use of Google Maps, and puts only modest requirements on the viewer's computer. In addition, we captured image stacks at different focal planes, and developed a web-based viewing system for three-dimensional navigation in the specimens. This novel technique is especially valuable for detailed visualization of large objects such as helminth eggs in stool specimens.

## Introduction

The Internet has made possible high standard educational undertakings with microscopy images also in the field of diagnostic medical parasitology (for an example, see: www.parasite-diagnosis.ch). However, the limitation until now has been that presentation of selected illustrations cannot replace working with a real microscope. The success of web-based virtual microscopy for histopatology [1] (www.webmicroscope.net) at the outset prompted us to demonstrate the histopathology of “the schistosome-infected mouse” which is presented in the beginning of this study. It soon became evident that some serious obstacles associated with education and quality control in medical parasitology can be solved using web-based microscopy, the main topic of this study.

Diagnostic parasitology, essentially being equivalent to microscopical examination of stool and blood samples, is performed globally at the basic level of the health care-system. Despite the recent introduction of polymerase chain reaction (PCR)-based methods, which make possible parasite identification also in cases of morphological identity, the methodology has changed little during the 150 years elapsed since it was described by Davaine [2],[3]. Fresh stool samples are studied under the microscope either as such or after the addition of Lugols solution to increase the sensitivity—and specificity—of the method. Additional concentration and staining procedures can be employed, but such procedures are usually performed at the next level, in parasitological laboratories associated with hospitals or microbiology departments of universities.

Whereas routine diagnostic methods in microbiology usually depend largely on cultivation under a variety of defined conditions under which microbial growth is quantified, diagnostic parasitology is equivalent to visual identification of parasites and/or parasite-derived materials. Thus, the quality of medical parasitology at the basic level relies heavily on the individual microscopist. Several good atlases describing medically important parasites have been published. The *Training Manual on Diagnosis of Intestinal Parasites* based on the World Health Organization (WHO) *Bench Aids for the Diagnosis of Intestinal Parasites* [4] has had a fundamental impact by providing a reference for the morphological identification of human parasites. Also ambitious external quality assessment programs have had a definitive effect, as shown by United Kingdom National External Quality Assessment Scheme (UKNEQAS) with a reported scheme of eight distributions a year to 285 participants [5]. However, several problems like differentiating between parasites and non-parasite material are difficult to solve. Thus, there is a need for developing quality assessment and education in parasitological diagnostics both in endemic and in non-endemic areas, based on defined samples containing helminth eggs and protozoa as well as “parasite-like” material, which may give false positive results. Besides distribution problems and challenges related to limited supply and costs involved, variation seriously restricts the widespread use of current quality assurance programs. Sample variation may depend on transportation and handling but the major problem is uneven distribution of parasites in the samples even if obtained from the same single source. To obtain defined and identical samples for large-scale distribution to several parasitology laboratories appears to be an unrealistic goal.

In the present report, by developing the Web Microscope for Parasitology (WMP), we have explored the potential of virtual microscopy to reach that goal. To identify parasites in suspensions it was necessary to develop a technique that facilitates navigation in the z-plane for “focusing”. In order to evaluate the potential of virtual microscopy to provide reference material for immunofluorescence (IF) microscopy, slides showing the staining patterns of specific antischistosome antibodies were also included. This was primarily motivated by the variation in published illustrations of the diagnostic schistosome gut reactive immune response seen in early infection [6].

## Materials and Methods

### Parasitological specimens

Paraffin sections of tissues from a *Schistosoma mansoni*-infected mouse [6] were stained with hematoxylin and eosin using standard protocols. Stool samples fixed in Sodium Acetate Acetic Acid Formalin (SAF) fixative, Giemsa-stained thick and thin blood films containing malaria parasites and a smear of a leishmaniasis skin lesion were from specimens sent for microscopical examination to the parasitology laboratory at the Swedish Institute for Infectious Disease Control (SMI; Stockholm, Sweden) and the Microbiology Department of the National University of Léon (UNAN; Léon, Nicaragua). Lugols iodine solution was used to enhance the microscopical features of protozoan cysts. Microscope slides were mounted under a cover slip for photography. For slides containing pools of several different stool parasites glycerol-gelatin was used as water-soluble mounting medium as described for insect specimens e.g. by Christie (www.psych.ubc.ca/bchristie/Techniques/PVA.htm) or Schauff (www.ars.usda.gov/SP2UserFiles/ad_hoc/12754100CollectingandPreservingInsectsandMites/collpres.pdf ).

For IF microscopy, we used sera from patients suffering from recent or chronic schistosomiasis and shown to exhibit typical serum antibody reactivity with the parasite. Frozen sections of *S. mansoni* adult worm pairs used for routine diagnostics were obtained by perfusion of infected mice as described previously [6]. Paraffin sections of worms fixed in Bouin's fixative gave essentially similar staining results, but were preferred as they were more stable.

### Digitization and introduction of parasitological specimens on Internet servers

Specimens were sent to the University of Tampere (Tampere, Finland) for digitization. The entire specimens on a microscope slide were scanned and photographed at high magnification (usually with a 40× objective), using a motorized microscope board. Sequentially acquired image tiles were stitched together, generating large image files in the order of 10–50 GB [1]. After image compression, the generated ‘virtual slides’ were uploaded to a network of image servers (www.webmicroscope.net/WMNetwork) for web-based viewing [1]. The ‘virtual slides’ can be viewed from personal computers with a web browser (MS Internet Explorer or Mozilla FireFox), allowing free navigation in the *x* and *y* planes at the desired magnification. Of note, in order to resemble real microscopy, a moderately fast Internet connection is required (e.g., 1–2 Mb/s and higher).

Regions of special interest (ROI) were indicated and some annotations added. The possibility to determine the size of an object is helpful especially in determining the size of intestinal protozoan cysts, but also to distinguish between parasite material and artefacts, such as pollen.

Pictures accompanying the text are screen images on the monitor seen at the Internet page (www.webmicroscope.net/parasitology). Images in the text were mounted using the Adobe Photoshop program.

## Results

**Figure 1:**
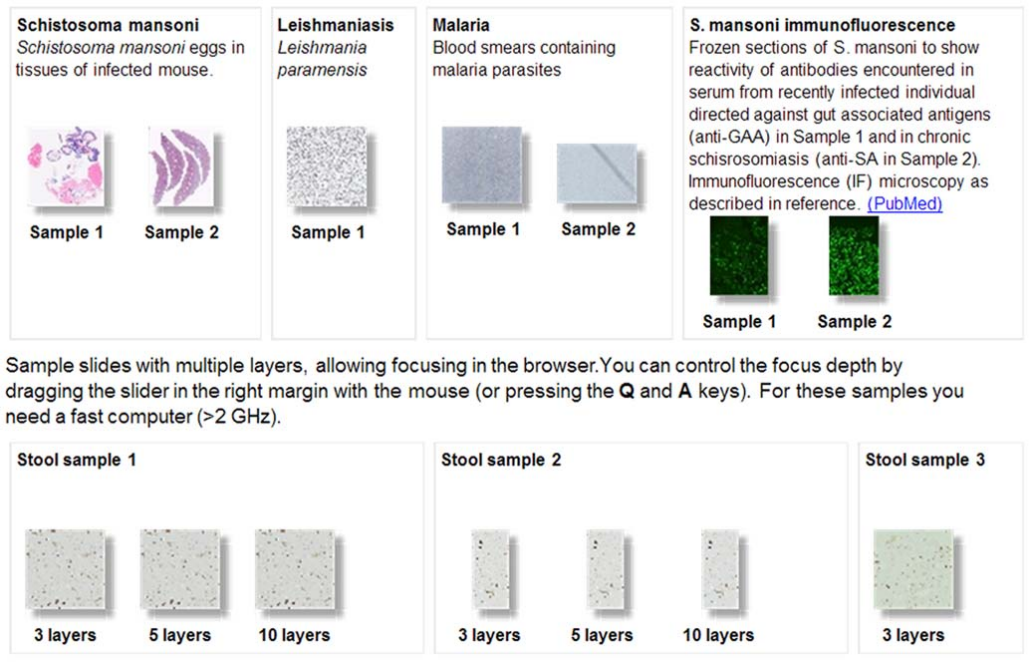
Part of the website for parasitology (www.webmicroscope.net/parasitology), showing “thumbnails” of currently available parasitological specimens (marked as *Sample*), which have been digitized for viewing at different magnification and freely navigable in the x and y plane. Focusing (navigation) in the z plane is possible in pictures made up of several layers as indicated for stool samples.

Ten specimens on microscope slides were digitized. They are seen in Figure 1 as “thumbnails” corresponding to part of the established website for medical parasitology (www.webmicroscope.net/parasitology). In line with the current use of virtual microscopy for histopathology, we have digitized tissue samples obtained from a mouse experimentally infected with *S. mansoni*.

**Figure 2:**
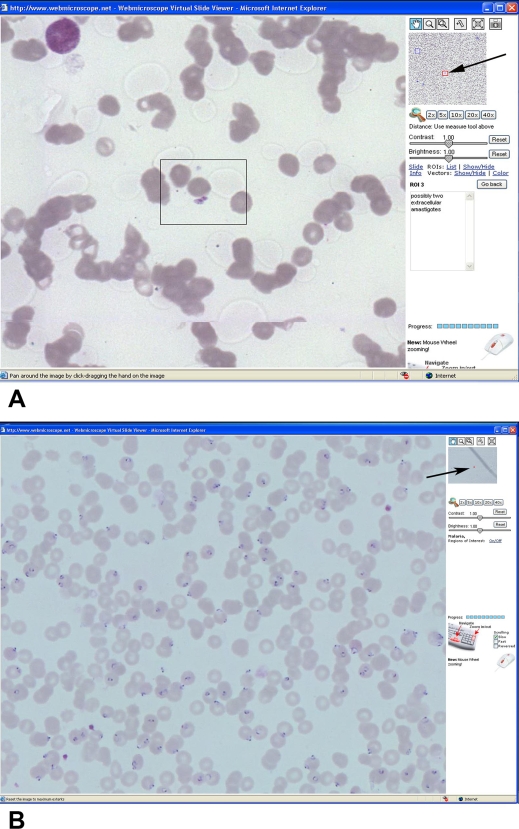
Smear from skin lesion containing *Leishmania* amastigotes in annotated area (ROI number 3). (A). Numerous plasmodia in thin blood film from patient with *Plasmodium falciparum* malaria of *Malaria Sample 2* (B). Giemsa-stained specimens at highest magnification (40× objective) as seen on the monitor screen.

We have digitized specimens containing some commonly occurring parasites. As “proof of principle” we show the appearance of malaria and leishmania parasites (Figure 2) and some helminth eggs and intestinal protozoan cysts in stool samples. For the latter specimens and to mimic actual microscopy, we developed a technique making “focusing” possible.

**Figure 3:**
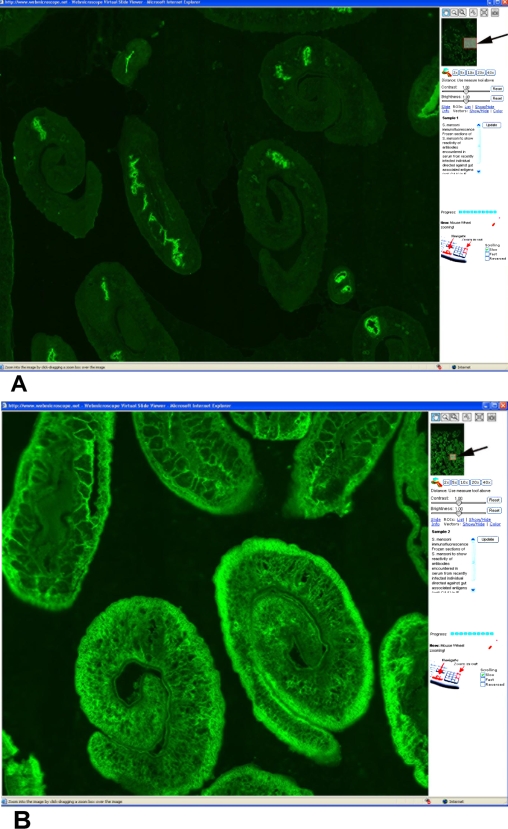
Two diagnostic staining patterns in the indirect IF test for antischistosoma antibodies using sections of adult *S. mansoni* worms as antigen. Antibodies (anti-GAA) react with “gut associated antigens” of male and female parasites (A). Antibodies (anti-SA) react with interstitial parenchymal “somatic” structures (B). The former pattern is typical for recently acquired infection whereas the latter pattern is seen in chronic infection. Magnification in A 5× objective, B 10×.

A further example of the use of virtual microscopy is to provide reference material for unusual, non-permanent or demanding parasitological assays. We show as an example, the diagnostics of acute schistosomiasis, which may depend on the demonstration of serum antibodies directed against excretory products of the intravascular worms. The IF samples showing serum antibody staining patterns typical for acute and chronic schistosomiasis, respectively on a number of sections of adult male and female schistosomes is seen in Figure 3.

**Figure 4:**
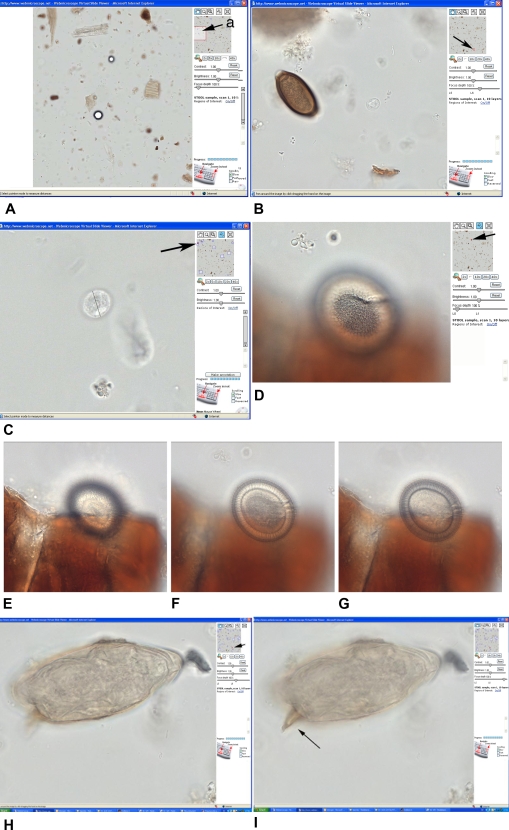
Helminth eggs and *Entamoeba* spp. cyst in “stool sample 1” at the website for parasitology (www.webmicroscope.net/parasitology). Arrows at the overview panes indicate the area of the samples, which are seen on the screen at the desired magnification. The size of any object can be measured using the ruler as seen in (C). The figures D to E show the appearance of a 3-dimensional object, a *Taenia* spp. egg, scanned at 4 different focal planes. The composite picture, which consists of 5 different layers, can be navigated in the z-plane which gives the illusion of smooth focusing. For further legends to this Figure 4 see Table 1.

The stool sample (“stool 1”, Figure 4) contains both helminth eggs and intestinal protozoan cysts, which can be examined at various magnifications: *Trichuris trichiura* (Figure 4B) and an *Entamoeba* cyst (Figure 4C). Fading of the iodine staining was evident in case of intestinal protozoa, whereas the stained appearance of *T. trichiura* eggs is due to endogenous material. The effect of increasing the number of layers is demonstrated. We can see that eggs in a stool sample scanned in three focal planes gives the illusion of focusing. However, the image obtained with more layers is superior, e.g., in examining the different structures of a *Taenia* spp. egg at various focal levels (Figures 4D–4G). Focusing of a *S. mansoni* egg shows the typical lateral spine (Figures 4H and 4I).

**Table 1:** Parasitological specimens at the WMP currently accessible at our website (www.webmicroscope.net/parasitology, see also Figure 1).

Specimen	Major findings	Comments
Tissues from *Schistosoma mansoni* infected mouse	Granulomas at different stages surrounding eggs in tissues.	Granulomas are seen around eggs passing through the intestinal wall (*Sample 1*) and around eggs trapped in the liver (*Sample 2*).
Smear from leishmaniasis skin lesion	*Leishmania panamensis*	Several parasites can be identified as probable *Leishmania* amastigotes. Figure 2A shows two of them.
Blood smears containing malaria parasites	*Plasmodium falciparum*	Two examples of stained blood films containing malaria parasites. *Sample 1* is an example of a technically unacceptable preparation with poor morphology of plasmodia. This contrasts with the technically excellent thin blood film seen in *Sample 2*, where erythrocytes containing early trophozoite (ring) stages are easily recognized. (Figure 2B)
*S. mansoni* worm pairs as antigen for specific serology	Distinct IF staining patterns are seen in serum specimens from acute and chronic schistosomiasis.	Serum antibodies in recently infected individual react with parasite-specific carbohydrates secreted from the intestinal epithelium of the worm (*Sample 1*). Figure 4. In chronic infection antibodies are directed against “interstitial”, parenchymal structures.
Stool samples	*Trichuris trichiura Taenia sp. Schistosoma mansoni Entamoeba* cysts	Three different stool samples have been scanned in 3–10 layers in order to enable “focusing”. Several helminth eggs and protozoan cysts are seen in the specimen area indicated in Figure 4: Examples of *Trichuris* (B), *Entamoeba* (C), *Taenia* (D, E, F), *S. mansoni* (H, J).

We observed that the “illusion of focusing” corresponds surprisingly well with the focusing in a real microscope. It also turned out that stool material covering a *Taenia* spp. egg does not interfere substantially with the resolution obtained in underlying structures. By adding up to 10 layers, the focusing capacity is increased further as expected, but data handling becomes somewhat slower. The use of several layers made it possible to readily identify protozoan cysts which are dispersed in the 3-dimensional space of the specimen.

The resolution using the 40× objective of our current microscope equipment is very good for helminth eggs and the focusing function obtained by scanning the specimen in two or more focal planes gives the authentic feeling of “focusing” like in an ordinary microscope. For intestinal protozoan cysts the resolution appears somewhat weaker, but still acceptable. The identification of, for example, some *Entamoeba* spp. cysts is possible in the specimen where the number of nuclei and their chromatin distribution is obvious.

## Discussion

Introduction of virtual microscopy of entire parasitological specimens as a new tool brings “virtual microscopy” very close to working with a real microscope. The author has lost the privilege of selecting “typical” microscopy fields for publication. Artefacts and non-parasite material, which can be mistaken for helminth eggs and intestinal protozoan cysts, cannot be ignored in digitized whole specimens. By presenting digitized whole parasitological specimens of different types at our website, we have illustrated these points.

Web microscopy for education and quality control in medical parasitology overcomes several problems basically due to the fact that parasites present in patient specimens are randomly distributed especially in suspensions. Thus, it is impossible to produce identical stool samples that are, for example, utilized to assess inter-laboratory agreement or for quality control studies (for example see [7]). As a result, quality assessment programs invariably lead to discussions on false negative and false positive results obtained by the participating centres. By using web-based virtual microscopy, we can distribute an identical sample and make it accessible worldwide via a network of image servers and discussions can be focused on observations on defined structures, which are traceable.

We conjecture that the basis for correctly assessing the disease burden and for measuring the effects of interventions relies on a somewhat shaky foundation as long as we do not know how reliable the reported laboratory findings are. The problem may be considerable in many developing countries. We simply do not know the fundamentals of false positive and false negative diagnostic findings, as we have seen in the case of “amebiasis” at health centers in Nicaragua [8]. Thus, the WMP may have an impact on the reliability of data, which constitute the basis for our understanding of the vast problem of neglected tropical diseases, a problem we have only started to explore [9].

By programming the motorized microscope stage to move also in the z-direction during digitization, 3–20 different focal planes were acquired for the specimens containing stool parasites. In the browser viewer the virtual slides corresponding to the focal planes can be stacked on top of each other in layers, ordered by their z-depth. By using a variable transparency when switching from one focal layer to another, an effect simulating focusing with a real microscope is created in the web browser.

If two focal layers are used in the browser, the amount of image data that needs to be loaded from the server is doubled. Addition of several layers would quickly saturate most network connections. We therefore developed a novel smart-focusing technique, which uses only a single focal layer when zooming or navigating in the x and y directions. However, immediately as the user moves in the z-direction (‘focuses’), either by ‘dragging’ a focusing slider on the web page, or pressing the focusing keys on the keyboard (Q and A), all available layers are turned on and begin loading data. Even if there are multiple layers, the required image data will load quickly as only the area of the specimen currently visible to the user needs to be refreshed. When the user again moves in the x or y direction or changes zoom-level, all but the currently visible layer are turned off, which enables faster zooming and panning.

With the smart-focusing method there is technically no limit on how many focal layers that can be added and stacked in the same browser window. In our tests we have found that up to 10 focal planes can be used, provided that the network connection is good (more than 2 Mb/s), and that the computer from which the slides are being viewed has a capacity of at least 2 GHz or a dual core processor. With ordinary office computers, connection speeds and monitor sizes, a maximum of 5 layers currently seems to be recommendable.

Several factors may influence the speed and smoothness of viewing virtual slides over a computer network. These include the bandwidth of the Internet connection, the end-user and server computer speed, as well as the size of the view area on the user's computer screen. The actual size of a virtual slide residing on the server does, however, not affect the viewing speed, since only the area currently visible on the user's screen is being processed and transmitted.

Larger image files, on the other hand, require more storage space on the image server. Within parasitology, the visualization and identification of protozoa would clearly be improved by using a higher objective and camera resolution in the scanning process. As the size of the produced virtual slides thus increases, longer digitization times are needed and the storage capacity needs grow exponentially. Especially if, in addition, multiple focal layers are captured at high resolution, data storage may currently be a rate-limiting factor. It may, however, be a temporary problem since storage costs are dropping rapidly and digitization speeds continuously improve.

The expanding global World Wide Web, faster communication speeds and the increase in number and capacity of personal computers, will clearly improve the usefulness of virtual microscopy. By incorporating a server into the local area network at SMI and the Karolinska Institutet (Stockholm, Sweden) we were able to view the virtual slides with high communication speed (∼100 Mb/s). Installation of a local server could be a solution also for hospitals or universities in developing countries with limited connections to the Internet. The technical requirements of a local server are modest and expenses could be kept low, but would clearly overcome data transfer problems. We have recently established a European Virtual Microscopy Network, which currently consists of virtual microscopy image servers and mirrored digital specimens in five European countries. This network automatically directs the user to the server with the best connection speed and according to an ongoing study, will further improve the image loading and viewing speed (unpublished data). Thus we think it is realistic to expect that WMP will benefit from further technical improvements and become a useful tool for parasitological education and quality control globally and we encourage proposals for utilizing the WMP within the framework of activities outlined above.
