# Correction: Web-Based Virtual Microscopy for Parasitology: A Novel Tool for Education and Quality Assurance

**DOI:** 10.1371/annotation/1f73ee39-9e3c-4ce4-9c35-2a6ab393de7d

**Published:** 2008-10-30

**Authors:** Ewert Linder, Mikael Lundin, Cecilia Thors, Marianne Lebbad, Jadwiga Winiecka-Krusnell, Heikki Helin, Byron Leiva, Jorma Isola, Johan Lundin

There was an error in affiliation 2 for authors Ewert Linder and Cecilia Thors. Affiliation 2 should be: 

2 Department of Microbiology, Tumor and Cell Biology (MTC), Karolinska Institutet, Stockholm, Sweden

